# Computed Tomography-Based Assessment of Transvalvular Pressure Gradient in Aortic Stenosis

**DOI:** 10.3389/fcvm.2021.706628

**Published:** 2021-09-08

**Authors:** Benedikt Franke, Jan Brüning, Pavlo Yevtushenko, Henryk Dreger, Anna Brand, Benjamin Juri, Axel Unbehaun, Jörg Kempfert, Simon Sündermann, Alexander Lembcke, Natalia Solowjowa, Sebastian Kelle, Volkmar Falk, Titus Kuehne, Leonid Goubergrits, Marie Schafstedde

**Affiliations:** ^1^Institute of Computer-assisted Cardiovascular Medicine, Charité – Universitätsmedizin Berlin, Berlin, Germany; ^2^DZHK (German Centre for Cardiovascular Research), Partner Site Berlin, Berlin, Germany; ^3^Department of Cardiology and Angiology, Charité – Universitätsmedizin Berlin, Berlin, Germany; ^4^Department of Cardiothoracic and Vascular Surgery, German Heart Center Berlin, Berlin, Germany; ^5^Department of Radiology, Charité – Universitätsmedizin Berlin, Berlin, Germany; ^6^Department of Cardiology, German Heart Center Berlin, Berlin, Germany; ^7^Department of Congenital Heart Disease, German Heart Center Berlin, Berlin, Germany; ^8^Einstein Center Digital Future, Berlin, Germany; ^9^Berlin Institute of Health (BIH), Charité – Universitätsmedizin Berlin, Berlin, Germany

**Keywords:** cardiac computed tomography, aortic stenosis, transvalvular pressure gradient, image-based modeling, reduced order model

## Abstract

**Background:** In patients with aortic stenosis, computed tomography (CT) provides important information about cardiovascular anatomy for treatment planning but is limited in determining relevant hemodynamic parameters such as the transvalvular pressure gradient (TPG).

**Purpose:** In the present study, we aimed to validate a reduced-order model method for assessing TPG in aortic stenosis using CT data.

**Methods:** TPG_CT_ was calculated using a reduced-order model requiring the patient-specific peak-systolic aortic flow rate (Q) and the aortic valve area (AVA). AVA was determined by segmentation of the aortic valve leaflets, whereas Q was quantified based on volumetric assessment of the left ventricle. For validation, invasively measured TPG_catheter_ was calculated from pressure measurements in the left ventricle and the ascending aorta. Altogether, 84 data sets of patients with aortic stenosis were used to compare TPG_CT_ against TPG_catheter_.

**Results:** TPG_catheter_ and TPG_CT_ were 50.6 ± 28.0 and 48.0 ± 26 mmHg, respectively (*p* = 0.56). A Bland–Altman analysis revealed good agreement between both methods with a mean difference in TPG of 2.6 mmHg and a standard deviation of 19.3 mmHg. Both methods showed good correlation with *r* = 0.72 (*p* < 0.001).

**Conclusions:** The presented CT-based method allows assessment of TPG in patients with aortic stenosis, extending the current capabilities of cardiac CT for diagnosis and treatment planning.

## Introduction

Aortic valve stenosis (AS) is the most common heart valve disease requiring surgery or intervention (1). In the aging population, the incidence of AS is increasing, reaching a prevalence of 12% in the population over 75 years of age (2). Patients with mild-to-moderate stenosis often remain symptom-free for a long period of time. However, mortality increases rapidly with the onset of symptoms (3). According to current guidelines, grading of AS severity is primarily based on the maximum flow velocity across the aortic valve, which is used for calculation of the transvalvular pressure gradient (TPG) and the aortic valve area (AVA) (4, 5).

CT became indispensable for planning of surgical or transcatheter aortic valve implantation (TAVI). This includes the assessment of anatomic structures, like the aortic annulus, the coronary ostia, and diameters and curvatures of all vessels from the catheter's entry side to the aortic valve. Moreover, CT contributes to the evaluation of AS severity by measurement of AVA (6, 7) as well as aortic valve calcium scoring in low-flow low-gradient AS (5, 8, 9). However, measurements of functional hemodynamic parameters, for example, TPG, that allow assessment of AS severity are not yet possible. Such functional measurements, which are currently only possible using echocardiography or cardiac catheterization, would improve the diagnostic value of CT in terms of a unified diagnostic tool.

The objective of this study is to introduce a method for TPG assessment using patient-specific aortic flow rates (Q) and AVA, both measured from cardiac CT images only. To validate this CT-based method, calculated TPG will be compared with catheter-based measurements.

## Methods

For this study, retrospective data of patients with AS treated with TAVI in two different centers between February 2019 and October 2020 were used. The following inclusion criteria were defined: aortic stenosis patients who were treated using TAVI; temporally resolved CT images were acquired for TAVI planning; and blood pressures in the left ventricle and the ascending aorta were invasively measured using catheterization during the TAVI procedure but before implantation of the device. No additional exclusion criteria were defined.

The study was registered at ClinicalTrials.gov (NCT04600739) and was approved by the institutional review board (IRB) (EA2/174/19). Individual informed consent was waived by the IRB due to the retrospective nature of this study.

### CT Image Acquisition

CT data sets of the entire heart were acquired as part of TAVI planning to assist in the choice of size and type of prosthesis. The median time between CT imaging and TAVI procedure was 12 (1–148) days. Following intravenous contrast material injection, an electrocardiogram-synchronized scan was performed using either a dual-source multislice spiral CT scanner (Somatom Definition Flash, Siemens Healthcare, Erlangen, Germany) or a wide area-detector volume CT scanner (Aquilion One Vision, Canon Medical Systems, or Revolution CT, GE Healthcare, Chicago, IL, USA). All scanning was performed at a tube voltage of 100 kV and an individually adapted tube current using the scanner exposure control software. For each subject, a multiphase data set was reconstructed that allows exact identification of the systolic phase with the widest aortic valve opening. All images were reconstructed with a standard soft-tissue convolution kernel and with the use of a dedicated noise-reduction software. Spatial and temporal resolution of the CT images varied. The spatial resolution used for segmentation was (0.39 – 0.648 mm) × (0.39 – 0.648 mm) in-plane resolution and (0.5–1 mm) slice thickness. The temporal resolution ranged from 70 to 140 ms.

### Measurement of Invasive Pressure Gradients

Based on the decision of the Heart Team, catheterization was performed under local (remifentanil) or general anesthesia (propofol and remifentanil). Blood pressure waveforms were measured in the ascending aorta as well as in the left ventricle using a 6-F pigtail catheter (Cordis, Dublin, Ireland). Measurements in the ascending aorta were always performed before measurements in the left ventricle. For each measurement, at least three consecutive cardiac cycles were acquired. The peak-systolic pressure in the left ventricle and the ascending aorta was calculated as the average of all peak values. TPG_catheter_ was then calculated as the difference between the average peak-systolic pressure in the left ventricle and the ascending aorta.

### Non-invasive Measurements of Pressure Gradients Using Doppler Ultrasound

In addition to the invasive measurement of TPG_catheter_, non-invasive measurements using Doppler ultrasound and the Bernoulli equation (TPG_echo_ = 4v^2^, where v is the maximum velocity) were acquired from clinical information systems. These measurements were performed as part of the preoperative diagnostic assessment within the same inpatient stay, but not simultaneously to either the catheterization or the CT image acquisition.

### Model for CT-Based Estimation of Pressure Gradients

**Figure 1:**
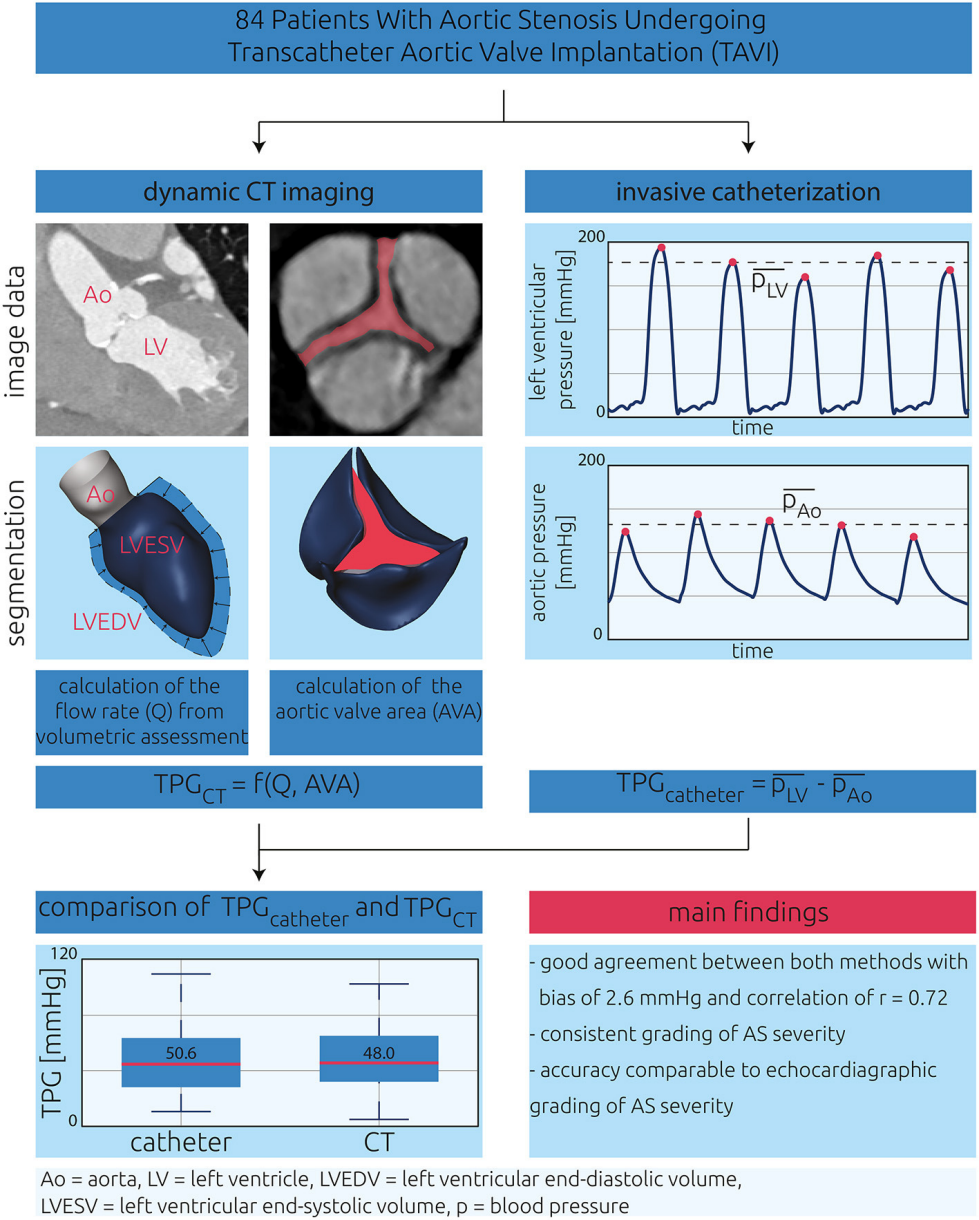
Central illustration describing the present study's design. The aim of this study was to validate a CT-based model for estimation of transvalvular pressure gradients (TPG_CT_). CT images and pressure waveforms of 84 patients with aortic stenosis were investigated in this retrospective study. TPG_CT_ was calculated using a reduced-order model requiring the aortic flow rate (Q) and the aortic valve area (AVA) as input. AVA was calculated by segmentation of the aortic valve leaflets, and Q was calculated from volumetric assessment of the left ventricle. For validation, invasively measured TPG_catheter_ was calculated from pressure measurements in the left ventricle as well as the ascending aorta.

A reduced-order model based on dynamic CT data was used for non-invasive assessment of TPG. For application of the model, the patient-specific volume flow rate (Q) passing through the stenosed aortic valve and the maximum AVA were measured using dynamic CT data (see Figure 1). First, the left ventricular volume was segmented for every time frame of the CT data using an approach described earlier (10, 11). Briefly, using an automated segmentation procedure, a predefined parametric model containing, among others, the left ventricle, the aortic bulbus, the ascending aorta, and the three aortic leaflets was adapted to the Hounsfield values of the patient-specific CT data for each time frame. Thus, the three-dimensional geometries of these anatomical structures were available. The peak-systolic volume flow rate was then calculated by calculating the left ventricular volume change between the respective time frames and dividing the difference by the temporal resolution of the CT images. In the presence of a mitral insufficiency (MI), Q was reduced by 10, 30, or 50%, for mild, moderate, and severe MI, respectively. Grading of MI severity was assessed from retrospective Doppler ultrasound reports. The regurgitation fraction used to adjust the aortic flow rate for MI corresponds to the lower thresholds defined for multimodal assessment of MI (12, 13).

The model used in the automatic segmentation procedure assumes an almost point-symmetric aortic valve, where all three leaflets are similar in size and shape. To mitigate this rather strong assumption, the aortic valve segmentation was manually adjusted to better represent the patient-specific valve. Here, a plane parallel to the aortic annulus was specified. Then, the contours of each leaflet were adjusted by manual interaction to fit on the Hounsfield contours visible in the CT images for multiple slices downstream the aortic annulus until the last leaflet tip was not visible anymore. This procedure was only performed for the peak-systolic phase where the maximum AVA was observed. AVA was calculated by identifying the minimal projected area enclosed by the three aortic valve leaflets.

With the use of both parameters, the non-invasive TPG_CT_ was calculated as

(1) $$\begin{array}{l} TPG_{CT}=185.5\cdot Q^{1.483}\cdot AVA^{-1.385} \end{array}$$

Here, TPG_CT_ is in mmHg, Q in l/s, and AVA in cm^2^.

For this study, the model's coefficients were adjusted compared with an initial feasibility study (11) as the segmentation procedure was optimized to better capture the asymmetry of native aortic valves. For the adjustment, computational fluid dynamic simulations were performed for 58 patients, following the description of Franke et al. (11). Thus, the numerical setup will only be described in a succinct manner.

The patient-specific anatomy of the left ventricle, ascending aorta, and aortic leaflets was smoothed; and the left ventricle was cut perpendicular to the left ventricular outflow tract's (LVOT's) centerline, ~20–30 mm below the aortic valve annulus. With the use of STAR-CCM+ (v. 15.02, Siemens PLM Software Inc., Plano, TX, USA), polyhedral volume meshes with a base size of 0.5–0.8 mm and a boundary layer consisting of five prism cell layers were generated. Each prism cell layer's thickness was 20% larger than that of the previous one. The overall thickness of this boundary layer was one-third of the base size. Near the aortic valve leaflets, a refinement region was specified, where the base size was reduced by half. At the LVOT, the peak-systolic volume flow rate Q was specified as boundary condition. At the end of the ascending aorta, a constant pressure outlet boundary condition was specified. Walls were assumed to be rigid, and a no-slip boundary condition was applied. Blood was modeled as non-Newtonian fluid with a constant density of 1,050 kg/m^3^. The fluid's dynamic viscosity was modeled using the Carreau–Yasuda model described by Karimi et al. (14). A realizable k-epsilon two-equation turbulence model was used. Simulations were considered converge if the calculated TPG was stable and convergence criteria for mass and momentum were below a threshold of 10^−3^. TPG was calculated as the difference in surface-averaged static pressure upstream and downstream the stenosed aortic valve. The three model coefficients, i.e., the constant coefficient as well the exponents for AVA and Q in Equation (1), were then calculated using the curve fitting toolbox and the non-linear least-square option provided by MATLAB (v. 2019b, MathWorks Inc., Natick, MA, USA).

Only data sets that were not used for validation (e.g., data of patients for whom a CT scan but no invasive pressure measurements were available) were included in this adjustment cohort.

### Statistical, Error, and Operator-Bias Analysis

Distributions of evaluated parameters were visualized using boxplots. Agreement between TPG_catheter_ and the TPG_CT_ was visualized using the Bland–Altman plots. Differences between heart rates during TAVI and CT as well as between TPG_catheter_ and TPG_CT_ were tested for significance. Here, the differences were tested for normal distribution using the Shapiro–Wilk test. In case of normal distribution, a paired-samples *t*-test was used. Otherwise, differences were tested using a Wilcoxon signed-rank test. The same metrics were also calculated for the agreement between TPG_catheter_ and TPG_echo_.

To investigate possible systematic error sources, the model's prediction error (TPG_catheter_ – TPG_CT_) was compared for the following confounding variables: HR difference between catheterization and CT, degree of MI, temporal resolution of the dynamic CT, and patient's sex. Here, a paired-sample *t*-test was used for normally distributed parameters, whereas a Mann–Whitney *U*-test was used otherwise. Additionally, some AS patients included in this study were identified as low-flow low-gradient cases. The prediction error of patients with low-flow low-gradient stenosis was then compared with the prediction error of those without. Here, a two-sample *t*-test was used.

For quantification of the intra- and interobserver variabilities, 10 cases were chosen from the validation cohort. For intraobserver variability, analysis segmentations of the aortic valve geometries were repeated by the main operator 6 months after the initial segmentations. In addition, segmentations were performed by another user for analysis of interobserver variability. Based on these segmentations, AVA was then calculated and compared with each other. For both analyses, intraclass correlation coefficients (ICCs) were calculated according to the convention by McGraw and Wong [ICC (1) (15)]. Only the operator bias on AVA was investigated, as the volume flow rate quantification was based upon the automatic segmentation of the left ventricle.

A significance level of *p* = 0.05 was used for all hypothesis tests.

## Results

Following the inclusion criteria, data of 84 (43 female) patients were used for validation of TPG_CT_. Patient characteristics at baseline are presented in Table 1. This information includes demographic, catheter-, CT-, and echocardiographic-based parameters. The average and standard deviation of AVA calculated from the segmented aortic valves was 0.75 ± 0.25 cm^2^, with individual values ranging from 0.31 to 1.77 cm^2^. The peak-systolic flow rate (Q) passing through the stenosed aortic valve featured an average and standard deviation of 296 ± 106 ml/s, with individual values ranging from 98 to 584 ml/s. TPG_CT_, which was calculated using Q and AVA, resulted in an average and standard deviation of 48.0 ± 25.9 mmHg.

**Table 1:** Overview of relevant parameters of the present study.

**Parameter**		**Mean ±*SD***	**Range**	**Data availability**	
**Name**	**Unit**		**Min**	**Max**	
**General patient information**					
Age	(Years)	82 ± 5	61	94	84 (100%)
Height^*^	(cm)	168 ± 10	145	195	78 (90%)
Weight^*^	(kg)	77 ± 19	35	135	80 (95%)
BSA^*^	(m^2^)	1.86 ± 0.25	1.20	2.41	78 (93%)
Systolic blood pressure (cuff measurement)^*^	(mmHg)	134.7 ± 22.8	90.0	198.0	76 (90%)
Diastolic blood pressure (cuff measurement)^*^	(mmHg)	71.0 ± 14.5	40	106	76 (90%)
**Computed tomography**					
AVA	(cm^2^)	0.79 ± 0.25	0.31	1.77	84 (100%)
Peak-systolic flow rate	(ml/s)	296 ± 106	98	584	84 (100%)
TPG_CT_	(mmHg)	48.0 ± 25.9	10.4	109.0	84 (100%)
Heart rate^*^	(1/min)	71 ± 20	37	132	81 (96%)
Number of time frames	(–)	20 (11–20)	6	22	84 (100%)
Ejection fraction	(%)	58 ± 15	19	83	84 (100%)
Stroke volume	(ml)	79.1 ± 24.9	25.9	138.7	84 (100%)
Stroke volume index^*^	(ml/m^2^)	43.3 ± 12.3	19.3	81.1	78 (93%)
Cardiac output	(l/min)	5.5 ± 1.8	2.6	11.7	84 (100%)
Total calcium volume^*^	(mm^3^)	986 ± 497	6	2,643	65 (77%)
At non-coronary leaflet	(mm^3^)	459 ± 292	0	1,298	65 (77%)
At left coronary leaflet	(mm^3^)	265 ± 150	0	781	65 (77%)
At right coronary leaflet	(mm^3^)	261 ± 177	0	1,085	65 (77%)
**Catheterization**					
Pressure in left ventricle	(mmHg)	162.7 ± 32.4	100	259	84 (100%)
Pressure in ascending aorta	(mmHg)	113.1 ± 25.0	65	182	84 (100%)
TPG_catheter_	(mmHg)	50.5 ± 28.0	5	160	84 (100%)
Heart rate^*^	(1/min)	63 ± 13	39	108	83 (99%)
**Echocardiography**					
${\text{TPG}}_{\text{echo}}^{*}$	(mmHg)	61.9 ± 22.0	20.0	118.0	80 (95%)
${\text{AVA}}_{\text{echo}}^{*}$	(cm^2^)	0.74 ± 0.17	0.4	1.1	74 (88%)
Stroke volume^*^	(ml)	52.2 ± 16.7	17.0	97.0	65 (77%)
Stroke volume index^*^	(ml/m^2^)	28.4 ± 9.0	9.3	52.5	63 (75%)
Ejection fraction^*^	(%)	57.3 ± 8.9	25.0	73.0	78 (93%)
Severity of aortic regurgitation (number of patients)^*^	(–)	None (11)	Mild (56)	Moderate (9)	76 (90%)
Severity of mitral regurgitation (number of patients)	(–)	None (9)	Mild (59)	Moderate (16)	84 (100%)

*All values, except for the number of time frames, are given as mean and standard deviation (SD). The number of time frames is specified as median and interquartile range (IQR) (75th−25th percentile). The range for all parameters is given as well. Body surface area (BSA) was calculated using the DuBois formula. Parameters highlighted using*

* *, i.e., echocardiographic information, except degree of mitral insufficiency, were not available for all patients. The number and percentage of available data sets are given as data availability in absolute values and percent*.

The average and standard deviation of the systolic, static pressure measured in the left ventricle and the ascending aorta was 162.7 ± 32.4 and 113.1 ± 25.0 mmHg, respectively, resulting in a TPG_catheter_ of 50.6 ± 27.4 mmHg. For TPG_echo_, the average and standard deviation was 61.9 ± 22.0 mmHg.

**Figure 2:**
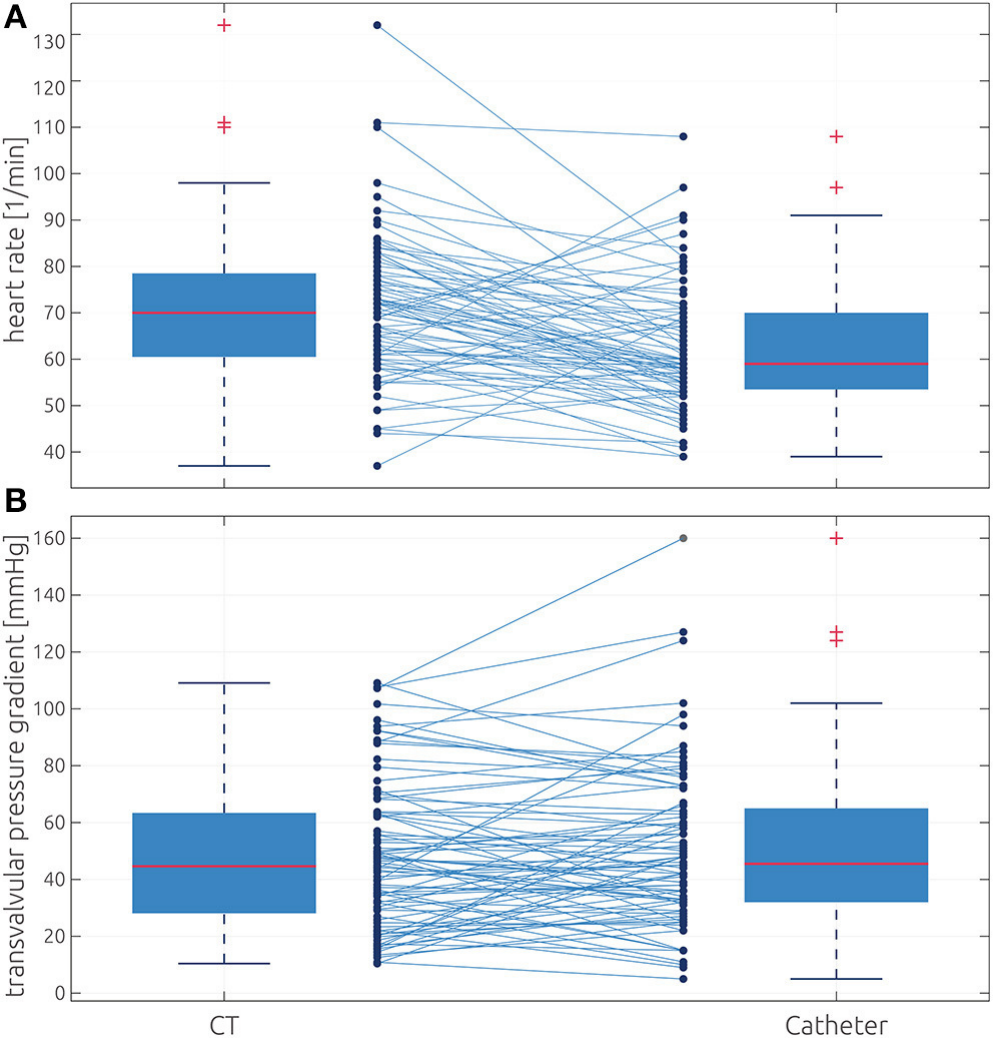
Comparison of heart rate and transvalvular pressure gradient distributions between both methods. **(A)** Box plots showing the measured heart rate during CT (left) and catheterization (right). **(B)** Box plots showing the distributions of TPG_CT_ (left) and the TPG_catheter_ (right). The box defines the range from the 25th to 75th percentile, whereas the whiskers indicate the inner 99% assuming normal distribution. Values outside this range are marked as outliers using red crosses. All individual values and their correspondence between both methods are shown as connected dots.

According to a Wilcoxon signed-rank test, the heart rate during catheterization was significantly lower than that during CT acquisition: 62.5 ± 13.2 vs. 70.7 ± 20.1 bpm, *p* < 0.001 (see Figure 2A). All individual parameters are provided as Supplementary Material.

### CT-Based Prediction of the Transvalvular Pressure Gradient

**Figure 3:**
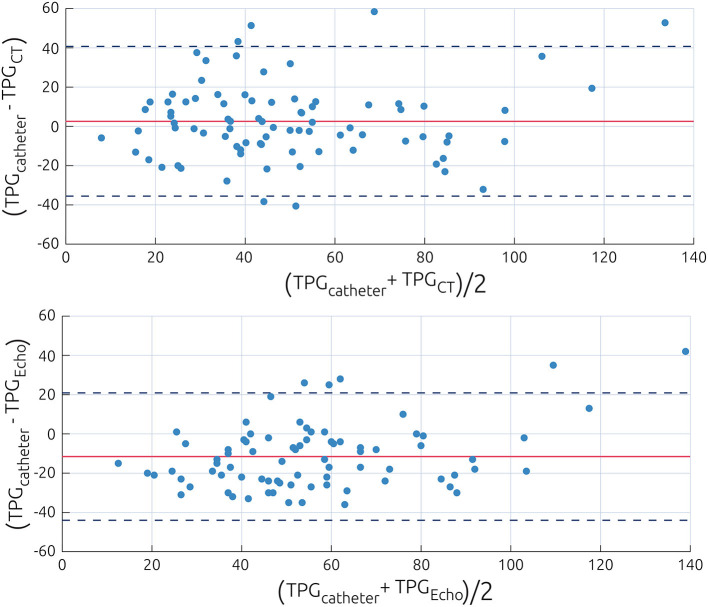
Bland–Altman plot visualizing the differences between the catheter-based measurements of the pressure gradient and the non-invasive estimations using the power law model **(Top)** or Doppler echocardiography **(Bottom)**. Data are presented as difference of both methods against the average of both methods. The value of the average prediction error is indicated by a solid line, whereas the dashed lines indicate the average prediction error ±1.96 times the standard deviation of this error.

**Figure 4:**
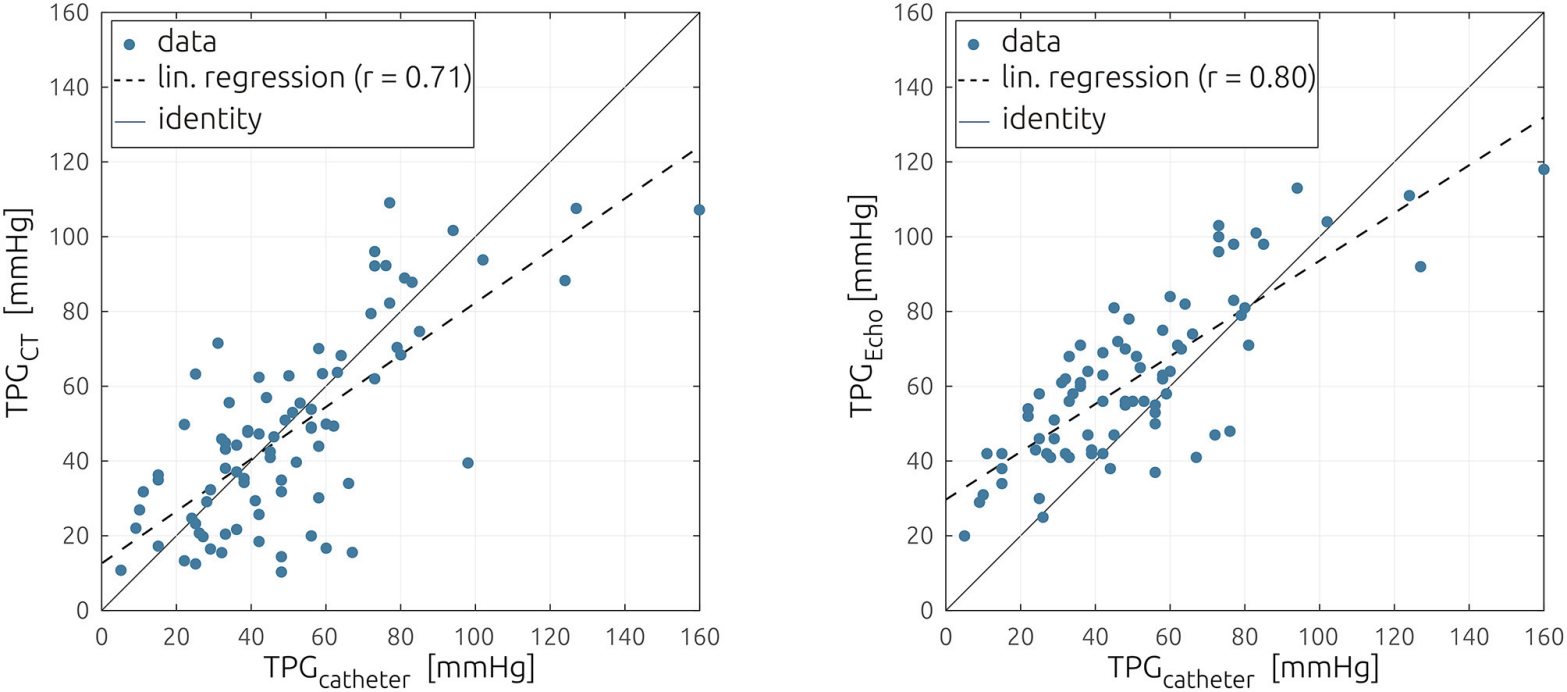
Scatter plot comparing the catheter-based measurements of the pressure gradient against the non-invasive estimation using the reduced-order model **(Left)** and echocardiography **(Right)**. The linear regression is shown as a dashed line.

A Bland–Altman plot comparing TPG_CT_ against TPG_catheter_ is shown in Figure 3. The bias between both methods, which is defined by the average of the differences of both methods and the standard deviation of those differences, was 2.6 ± 19.3 mmHg, which resulted in relatively wide limits of agreement, defined as mean ± 1.96 times the standard deviation of −35.2 to 37.9 mmHg. The means of both methods did not differ significantly, according to a Wilcoxon signed-rank test (*p* = 0.56), and a good correlation between the non-invasive estimation of TPG and the catheter-based measurements was observed (*r* = 0.72, *p* < 0.001, see Figure 4). Both pressure gradient distributions and all individual values of both methods are shown in Figure 2B. In contrast, the correlation between TPG_catheter_ and TPG_echo_ was *r* = 0.80, and the average and standard deviation of the differences between those two methods was −11.5 ± 16.4 mmHg, resulting in limits of agreement of −43.6 to 20.6 mmHg. TPG_echo_ systematically overpredicted TPG_catheter_ (*p* < 0.001, paired-samples *t*-test).

Discriminating patients by their sex revealed only minor differences in the predictive capabilities of the model. For women, the average and standard deviation of the differences between both methods was 5.2 ± 18.8 mmHg whereas −0.1 ± 19.5 mmHg for men (*p* = 0.14). Significant differences between women and men were found for AVA (0.71 vs. 0.87 cm^2^, *p* < 0.001) and volume flow rate (271 vs. 321 ml/s, *p* = 0.03), with both parameters being larger in men than in women. While TPG_catheter_ (55.4 vs. 45.7 mmHg) and TPG_CT_ (50.2 vs. 45.8 mmHg) were larger in female patients than in male patients, these differences were not significant (*p* = 0.11 and 0.47, respectively).

**Figure 5:**
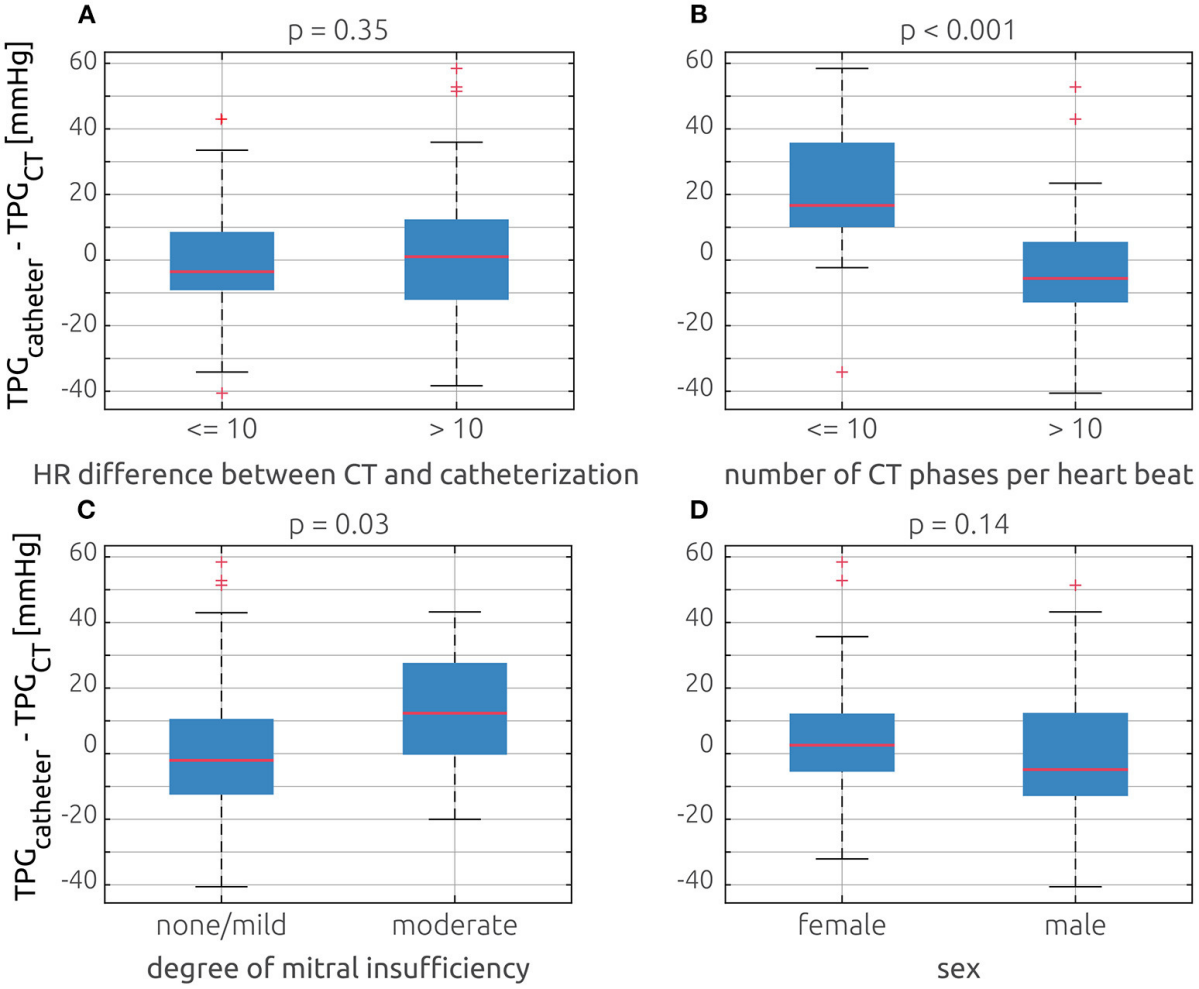
Comparison of the model's prediction error (TPG_catheter_ – TPG_CT_) based on four different possible error sources using box plots: **(A)** comparison of patients who have absolute heart rate difference between catheterization and CT equal or below 10 bpm and patients whose difference exceeded that threshold. **(B)** Comparison of cases, where the dynamic CT had 10 or less phases per heartbeat and those with more phases. **(C)** Comparison of patients who had no or a mild mitral insufficiency and those with moderate insufficiency. **(D)** Comparison between female and male patients.

Additional to the effect of the patients' sex, the influence of three possible sources of error on the accuracy of the model was investigated. No relevant bias in the model's accuracy was found between patients who featured an absolute heart rate difference between CT and catheterization of 10 bpm or more and those patients with similar heart rates during both procedures: 3.2 ± 21.0 vs. −1.1 ± 17.3 mmHg, *p* = 0.35. In contrast, the temporal resolution of the dynamic CT images has a strong effect on the model's prediction error. The average and standard deviation of the prediction error was 21.3 ± 20.9 mmHg for cases where a heartbeat was resolved with 10 phases or less, whereas it was −3.4 ± 16.2 mmHg when a higher temporal resolution was available (*p* < 0.001). The model's prediction error was also larger in patients with moderate MI compared with patients with no or only mild MI: 12.1 ± 18.1 vs. 0.17 ± 20.2 mmHg, *p* = 0.03. The respective distributions of the model's prediction error are shown in Figure 5. Finally, the prediction error for patients who have been identified as low-flow low-gradient cases (*n* = 11) during clinical routine was compared with that of all other patients (*n* = 63). On average, in low-flow low-gradient cases, TPG_CT_ was larger than TPG_catheter_ (−8.6 ± 15.0 mmHg), whereas predicted TPG_CT_ was smaller than the catheter-based measurements for all other patients (4.3 ± 19.6 mmHg). However, this difference was not significant according to a two-sample *t*-test (*p* = 0.25).

To quantify consistency in grading of AS severity, a maximum TPG threshold of 64 mmHg was used, corresponding to the maximum velocity of 4 m/s and the corresponding mean threshold of 40 mmHg defined in clinical guidelines. In 77 of 84 patients (91.7%), both methods consistently predicted a TPG of either below or above 64 mmHg. In four patients (4.8%), TPG_catheter_ was above this threshold, while TPG_CT_ was smaller than 64 mmHg. Finally, in two patients (2.3%), TPG_CT_ was above 64 mmHg, whereas TPG_catheter_ was below this threshold.

ICCs calculated for the intra- and interobserver analyses were 0.99 and 0.88, indicating an excellent intraobserver reliability and a good agreement between different observers.

## Discussion

Our results show that CT-based estimation of TPG performs well compared with invasive cardiac catheterization. The proposed method is easy to apply and complements the current diagnostic capabilities of cardiac CT imaging by providing additional functional hemodynamic information.

### CT-Based Analysis of Cardiac Anatomy and Function

Recent advancements in cardiac CT have led to significant improvement in image quality while exposure to radiation simultaneously decreased (16, 17). As a result, CT became an essential tool for treatment planning in AS. In clinical routine, CT is mainly used for the evaluation of anatomical structures of the aorta, the valve annulus, coronary arteries, and the LV outflow tract. However, the valve's geometry can also be visualized using CT, allowing direct measurement of AVA (7, 18). The additional possibility of grading the severity of AS using TPG would further enhance the value of CT by transforming it to a unified diagnostic tool for the evaluation of structural heart disease, combining comprehensive assessment of anatomic and hemodynamic information. The presented method is straightforward and allows assessing TPG from ventricular volumes and the anatomy of the aortic valve. While complete automation of the segmentation of the patient-specific left ventricle and the aortic valve is possible (10), manual correction was still necessary due to the aortic valve's asymmetry. However, this correction could also be automated in the future, so that a fully automated approach might be feasible.

The ability to determine hemodynamic parameters would complement various other CT-based functional measurements that have been established in recent years for cardiovascular applications. Well-known examples are measurement of myocardial perfusion (19, 20) or calculation of fractional flow reserve in patients with coronary artery disease (21–23).

### Grading of Aortic Stenosis Severity

According to clinical guidelines, grading of AS severity is required for treatment planning (4, 5). This grading is currently based on either AVA or TPG. Except for planimetric assessment of AVA, models are used to calculate these parameters from either the velocity information obtained using Doppler echocardiography (e.g., Bernoulli equation for TPG; continuity equation for AVA) or the pressure obtained during heart catheterization (e.g., Gorlin equation for AVA). However, those models require additional measurements, for example, cross-sectional areas of the LVOT and the aorta for application of the continuity equation or the cardiac output and systolic ejection period for application of the Gorlin equation, increasing their uncertainty. For both echocardiography- and catheter-based classifications, inconsistent gradings of AS severity are reported in ~30% of patients (24, 25).

Due to its ubiquitous availability and non-invasiveness, echocardiography is the standard for diagnosis of patients with AS (12), even though it is known to systematically overpredict TPG compared with catheterization (26). This overestimation results from neglect of blood flow velocities distal to the aortic valve by the Bernoulli equation. Furthermore, the method is susceptible to interobserver variability (27). However, in a well-controlled environment, where echocardiographic and catheter-based assessments of AS severity are performed simultaneously, good correlations (*r* > 0.9), low standard errors of estimate (SEEs <15 mmHg), and good agreement for graduation of AS severity between both methods were reported (28–31). In contrast, performing the non-invasive assessment 1 week before catheterization led to slightly weaker correlations (*r* ≈ 0.8) and increased errors (SEE ≈ 25 mmHg) (30–32).

In our study, we also found a slight overestimation of echocardiography based TPG_echo_. However, TPG_echo_ correlated well with TPG_catheter_. While the Bland–Altman analysis revealed relatively large limits of agreement, those were similar to previous studies comparing echocardiography and catheterization. Thus, good agreement with respect to the expected accuracy, except for the systematic overestimation, was found. In terms of the agreement between TPG_CT_ and TPG_catheter_, our results were also similar to the findings reported in studies comparing invasive catheterization and echocardiography as well as the echocardiographic measurements acquired for this study's cohort. With a consistent grading of severity in 92% of all cases, the presented approach performed exceptionally well in this regard.

In this study, direct measurements of TPG using cardiac catheterization, clinically considered as gold standard, was considered as ground truth against which the reduced-order model was validated. However, even this technique is associated with errors. Using two independent measurements in the LV and the aorta rather than simultaneous pressure measurements may affect the quantification of TPG. Furthermore, the position of the catheter in the ascending aorta, as well as the additional decrease of the aortic valve's cross section due to the catheter, especially in patients with already low AVA, may affect accuracy (33).

### Error Analysis

Analysis of different probable sources for bias revealed that the model's prediction of TPG worked equally well for male and female patients. While a heart rate difference between CT and catheterization of 10 bpm or more resulted in significantly larger prediction error, this effect was rather small. The presence of a moderate MI as well as a limited temporal resolution of CT images of 10 or less phases resulted in differences in the model's prediction error of more than 10 mmHg on average. As the volume flow rate Q is derived from left ventricle segmentations, the limited temporal resolution resulted in an inaccurate assessment of the left ventricle volume change and thus of the volume flow rate. Therefore, the peak-systolic flow rate was underestimated, which led to an underestimation of TPG_CT_.

Similarly, TPG_CT_ was smaller than TPG_catheter_ for patients with moderate MI, suggesting that the compensation of the aortic flow was too rigorous, resulting in an underestimation of Q and thus of TPG_CT_. If the model is only applied to the 55 patients with a temporal CT resolution of more than 10 phases per heartbeat and no or only a mild MI, the correlation between methods increases to *r* = 0.81, and SEE decreases to 15.3 mmHg. Out of the seven outliers shown in the Bland–Altman plot (Figure 3), five had a moderate regurgitation, or a limited temporal resolution, or both. Therefore, more thoroughly constraining the inclusion criteria for future investigations using the presented approach might be warranted.

### Limitations

There are several important limitations to this study. Due to the retrospective study design, data acquisition was not performed simultaneously but with a median delay of 12 (1–148) days, which certainly decreases the agreement between all methods compared. In contrast to CT and transthoracic echocardiographic (TTE) examinations, catheterization was performed under sedation, which further affects hemodynamics and therefore accuracy of TPG assessment. Additionally, the proposed CT-based TPG assessment still requires minor manual interaction, which might represent a potential additional error source.

As the study only includes TAVI patients, we cannot exclude a certain selection bias due to the type of intervention. However, due to the retrospective study design, this limitation could not be mitigated, as TAVI patients were the only AS patients for whom pressure gradients were routinely measured using catheterization. Also, the decision between TAVI and surgical valve replacement is based on risk scores rather than the severity of the AS; thus, neither the AVA nor the peak-systolic volume flow rate is relevant for this decision. The stenosis relevant parameter (e.g., AVA and TPG) distributions are similar between patients undergoing TAVI or surgical valve replacement (34). Nonetheless, the validity of this assumption as well as the applicability of this approach in patients with low gradients should be evaluated in a future prospective trial.

### Outlook

The presented reduced-order model might be extended by a functionality to also predict TPG during physical exercise in the future. The change of cardiovascular functional parameters (e.g., cardiac output or peak-systolic flow rate) due to physical exercise can be predicted if these parameters were known at rest (35). In our own preliminary work comparing cardiac catheterization and pharmaceutical exercise testing using dobutamine, the increase in TPG during physical stress was successfully predicted (36). As changes in AVA during physical stress are rather small, this approach might be applicable to our approach, allowing non-invasive assessment of TPG at different levels of exercise. The findings of this study, especially regarding the level of agreement between both methods and possible error sources, are vital for the design of a prospective validation study. Here, an *a priori* power analysis for sample size estimation, simultaneous acquisition, or at least acquisition in close succession of CTA and catheter-based pressure gradients seems warranted.

## Conclusion

The presented approach allows estimation of TPG in patients with AS from dynamic cardiac CT images only. This additional hemodynamic assessment might complement the current diagnostic capabilities of cardiac CT and hence the preoperative treatment planning of patients with AS.

## Data Availability Statement

The original contributions presented in the study are included in the article/Supplementary Material, further inquiries can be directed to the corresponding author/s.

## Ethics Statement

The studies involving human participants were reviewed and approved by Ethikkommission der Charité—Universitätsmedizin Berlin. Written informed consent for participation was not required for this study in accordance with the national legislation and the institutional requirements.

## Author Contributions

LG, TK, and MS: conceptualization. HD, AB, AL, NS, and BJ: data curation. BF, JB, and LG: formal analysis and methodology. TK and MS: funding acquisition and supervision. MS, HD, AB, AL, and BJ: investigation. JB and MS: validation. BF and JB: visualization. BF, JB, and MS: writing—original draft. All authors writing—review and editing. All authors contributed to the article and approved the submitted version.

## Funding

MS was participant in the BIH-Charité Junior Digital Clinician Scientist Program funded by the Charité—Universitätsmedizin Berlin and the Berlin Institute of Health.

## Conflict of Interest

The authors declare that the research was conducted in the absence of any commercial or financial relationships that could be construed as a potential conflict of interest.

## Publisher's Note

All claims expressed in this article are solely those of the authors and do not necessarily represent those of their affiliated organizations, or those of the publisher, the editors and the reviewers. Any product that may be evaluated in this article, or claim that may be made by its manufacturer, is not guaranteed or endorsed by the publisher.
